# DNA methylation and gene expression of *HIF3A*: cross-tissue validation and associations with BMI and insulin resistance

**DOI:** 10.1186/s13148-016-0258-6

**Published:** 2016-09-02

**Authors:** Ailsa Maria Main, Linn Gillberg, Anna Louisa Jacobsen, Emma Nilsson, Anette Prior Gjesing, Torben Hansen, Oluf Pedersen, Rasmus Ribel-Madsen, Allan Vaag

**Affiliations:** 1 Department of Endocrinology, Rigshospitalet, Section 7652, Tagensvej 20, DK-2200 Copenhagen, Denmark; 2 Faculty of Health and Medical Sciences, University of Copenhagen, Copenhagen, Denmark; 3 Department of Clinical Sciences, Lund University Diabetes Centre, Malmö, Sweden; 4 Section of Metabolic Genetics, The Novo Nordisk Foundation Center for Basic Metabolic Research, Faculty of Health and Medical Sciences, University of Copenhagen, Copenhagen, Denmark; 5 The Danish Diabetes Academy, Odense University Hospital, Odense, Denmark

**Keywords:** Epigenetics, Obesity, Type 2 diabetes, Heritability, Insulin sensitivity

## Abstract

**Background:**

Associations between BMI and DNA methylation of hypoxia-inducible factor 3-alpha (*HIF3A*) in both blood cells and subcutaneous adipose tissue (SAT) have been reported. In this study, we investigated associations between BMI and *HIF3A* DNA methylation in the blood and SAT from the same individuals, and whether *HIF3A* gene expression in SAT and skeletal muscle biopsies showed associations with BMI and insulin resistance. Furthermore, we aimed to investigate gender specificity and heritability of these traits.

**Methods:**

We studied 137 first-degree relatives of type 2 diabetes (T2D) patients from 48 families, from whom we had SAT and muscle biopsies. DNA methylation of four CpG sites in the *HIF3A* promoter was analyzed in the blood and SAT by pyrosequencing, and *HIF3A* gene expression was analyzed in SAT and muscle by qPCR. An index of whole-body insulin sensitivity was estimated from oral glucose tolerance tests.

**Results:**

BMI was associated with *HIF3A* methylation at one CpG site in the blood, and there was a positive association between the blood and SAT methylation levels at a different CpG site within the individuals. The SAT methylation level did not correlate with *HIF3A* gene expression. Interestingly, *HIF3A* expression in SAT, but not in muscle, associated negatively with BMI and whole-body insulin resistance. We found a significant effect of familiality on *HIF3A* methylation levels in the blood and *HIF3A* expression levels in skeletal muscle.

**Conclusions:**

Our findings are in line with the previously reported link between BMI and DNA methylation of *HIF3A* in the blood. The tissue-specific results of *HIF3A* gene expression indicate that SAT is the more functional tissue in which a low expression may adversely affect whole-body insulin sensitivity.

**Electronic supplementary material:**

The online version of this article (doi:10.1186/s13148-016-0258-6) contains supplementary material, which is available to authorized users.

## Background

*HIF3A* belongs to the transcription factor family of hypoxia-inducible factors (HIFs) which regulate the cellular response to hypoxia [1]. HIFs are dimers of an α-subunit (HIF-1α, HIF-2α, or HIF-3α) and a ß-subunit where complexes of HIF-3α are thought to oppose the actions of those formed by HIF-1α and HIF-2α [1, 2]. It has been shown that adipose tissue-specific *Hif3a* knockout mice are resistant to weight gain and have a better glucose tolerance and insulin sensitivity [3]. HIF-3α is highly expressed in adipocytes and acts as an accelerator of adipogenesis [4] and may also be involved in the regulation of glucose metabolism since it is upregulated by both insulin and 2-deoxy-d-glucose-induced glucoprivation [5].

The etiology of type 2 diabetes (T2D) consists of both genetic and environmental factors [6]. One mechanism whereby environmental factors can contribute to T2D is epigenetics. Epigenetics is the study of heritable changes in DNA that affect gene transcription irrespective of the DNA sequence, such as methylation of DNA cytosine residues (mainly CpG sites) [7]. Twin studies indicated that both environmental and heritable factors affect epigenetic modifications [8]. Other studies have shown that epigenetic patterns can be changed by age [9, 10] and environmental factors such as diet and exercise [11–14]. Methylation of gene promoter regions can lead to silencing of gene expression whereas DNA methylation in intron-spanning regions of the gene body may result in alternative splicing [15].

A genome-wide study of DNA methylation found positive associations between BMI and DNA methylation levels of three sites in the first intron of *HIF3A* in whole blood from 479 individuals of both genders and in subcutaneous adipose tissue from 635 women [16]. Aside from the relation between methylation status and BMI, these results also underline the potency in whole blood DNA methylation profiling as a marker for epigenetic changes in other human tissues [9, 16]. For one of the investigated CpG sites, the methylation level was inversely associated with *HIF3A* gene expression in adipose tissue. Recently, other studies replicated the findings of BMI-associated DNA methylation of *HIF3A* in the blood from men and women [17, 18] and in adipose tissue where the association was only significant in women [9].

In this study of 137 first-degree relatives of T2D patients from 48 families, our aim was to determine whether *HIF3A* methylation in the blood and SAT, and *HIF3A* gene expression in SAT and skeletal muscle, are associated with BMI and whole-body insulin sensitivity. Furthermore, we aimed to investigate if these associations are gender specific and whether these traits are heritable.

## Methods

### Study design and population

One hundred and thirty-seven Danish individuals (51 men and 86 women) from 48 different families were recruited in 2005–2007 as part of the EUGENE2 Consortium study population [19]. They were all first-degree relatives of patients with T2D. The subjects were recruited regardless of their own current glucose tolerance status. In total, there were 48 families represented by one member (*n* = 13), two (*n =* 12), three (*n =* 10), four (*n =* 5), five (*n =* 4), six (*n =* 2), eight (*n =* 1), or ten members (*n =* 1). Twenty-six individuals had T2D of which nine received insulin treatment and were asked to discontinue their treatment 12 h in advance of the clinical examination [19]. The study was approved by the Ethical Committee of the Capital Region of Denmark (KA 05041g, 21-04-2005). All participants signed a consent form after written and oral information.

### Clinical examinations

A standard 75-g oral glucose tolerance test (OGTT) was performed in the morning in the fasting state [19] in all participants, and blood samples were drawn before and 30, 60, and 120 min after ingestion of the glucose load. Plasma glucose and insulin levels were measured in all samples. Glucose tolerance status was determined based on WHO guidelines [20]. Whole-body insulin sensitivity was estimated from fasting plasma glucose and insulin levels by calculating the Matsuda insulin sensitivity index [19, 21].

Subcutaneous adipose tissue (SAT) biopsies from the abdomen and skeletal muscle biopsies from the vastus lateralis muscle were taken with a Bergström needle, snap frozen in liquid nitrogen, and stored at −80 °C until analysis [19]. For both biopsies, Xylocain (20 mg/ml) was used as a local anesthetic.

### DNA methylation analysis

Genomic DNA was extracted from SAT using QIAamp DNA Mini Kits (Qiagen, Hilden, Germany) and from whole blood using blood QIAamp DNA Blood Mini Kits (Qiagen)

**Fig. 1:**
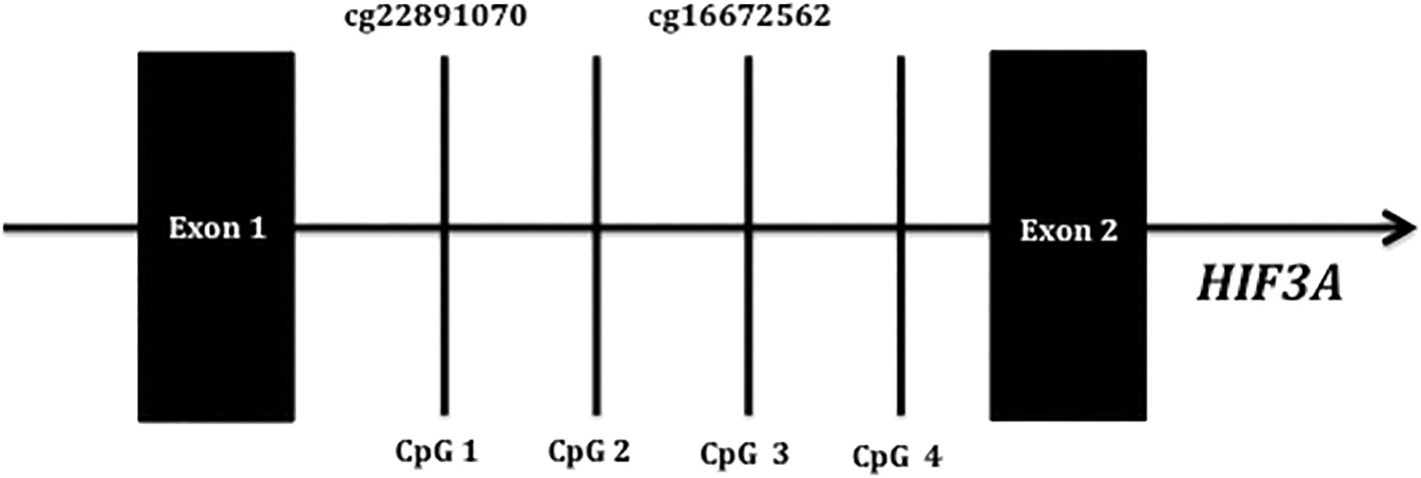
The four CpG sites located between exon 1 and exon 2 of *HIF3A* approximately 1,340 bp downstream from the transcription start site

The EpiTect 96 Bisulfite Kit (Qiagen) was used for the bisulfite conversion of 400 ng genomic DNA from whole blood and SAT. The level of methylation of four CpG sites located between the first and second exon in *HIF3A* (Fig. 1) was determined by pyrosequencing of bisulfite-treated DNA from the blood (*n =* 136) and SAT (*n =* 137). Primer assays were designed using the Pyromark Assay Design 2.0 software (Qiagen) (forward primer 5′-TTTTGGTTTTGGGTTTAATAAGGAA-3′, reverse primer 5′-biotin-AAAAAAAATATTAAAAACCCACTCACC-3′, sequencing primer 5′-GGTGTTTTTTTTTTTTATTTAAGGT-3′). This primer set covered two sites previously investigated by Dick et al. (CpG site 1: cg22891070 and CpG site 3: cg16672562) and two additional sites (CpG sites 2 and 4) (Fig. 1). A third site (cg27146050) was previously investigated by Dick et al. [16], and to investigate this site, we used a different primer set. However, our results on this site were not of prime quality, possibly due to lack of primer specificity, and were thus not included in the present study.

The PyroMark PCR kit (Qiagen) was used to amplify the bisulfite-converted DNA according to the manufacturer’s protocol, and PCR amplicons were visualized after electrophoresis through a GelRed-stained 3 % agarose gel. The PyroMark Q96 Vacuum Workstation (Qiagen) was used for preparation of the samples, and pyrosequencing was performed with the Pyromark Q96 ID Instrument (Qiagen). The data was analyzed using the PyroMark Q96 software v.2.5.8.15 and validated manually. Samples with unreliable methylation results were re-run (uncertainties due to baseline shift, low signal-to-noise ratio, low peak height, and large peak height deviation at positions that were close to the CpG sites analyzed). All DNA methylation results from the re-run that did not meet the quality criteria were excluded. The final analysis is based on 105–108 individuals in the blood and 83–84 individuals in SAT (Additional file 1: Table S1).

### Gene expression analysis

Total RNA from SAT (*n =* 137) was extracted with the miRNeasy Kit (Qiagen) and converted to complementary DNA (cDNA) by the High Capacity cDNA Reverse Transcription Kit (Applied Biosystems, Grand Island, NY, USA). Total RNA was extracted from muscle tissue (*n =* 129) using TRI Reagent (Sigma-Aldrich, St. Louis, MO, USA) and converted to cDNA using the QuantiTect Reverse Transcription Kit (Qiagen) [19]. The *HIF3A* gene expression in both tissues was determined by quantitative real-time PCR on a ViiA 7 Real-Time PCR System (Applied Biosystems, Foster City, CA, USA) using SYBR® Green RT-PCR Reagents Kit (Life Technologies, Grand Island, NY, USA) and primers specific for *HIF3A* messenger RNA (mRNA) (NM_152794, forward primer: 5′-CTTTCTGCTCTTTCCTCTCAGC-3′, reverse primer: 5′-GCTCATTCAGGTTCAGGAGTG-3, Tag Copenhagen, Copenhagen, Denmark). The *HIF3A* mRNA quantity was normalized to the mRNA expression of the housekeeping gene *HPRT1* (forward primer: 5′-TGACCTTGATTTATTTTGCATACC-3′, reverse primer: 5′-CGAGCAAGACGTTCAGTCCT-3′). Each sample was run in duplicates, and we used the ΔΔC_t_ method for quantification of mRNA levels. mRNA results with C_t_ values above 33 cycles or a C_t_ difference >0.35 on duplicates were re-run (SAT *HPRT1*: 34 samples; SAT *HIF3A*: 0; skeletal muscle *HPRT1*, *HIF3A*: 0), and samples still exceeding the cut-offs after re-analysis were excluded. The final analysis is based on 117 individuals in SAT and 120 individuals in skeletal muscle (Additional file 1: Table S1).

### Statistical analysis

The data was analyzed by a linear mixed model in R version 3.1.0 (http://www.r-project.org) with family number as a random factor and sex, age, BMI, and HbA1c as fixed factors in all models. All components of the mixed model were checked for distribution normality by evaluation of histograms. Factors that did not show normal distribution were transformed by natural logarithm. Residuals from the mixed model analyses were checked for normality by qq-plots. Furthermore, all analyses were run separately for each sex and without inclusion of T2D patients. The results from the mixed models are presented as *β* (effect estimate) with 95 % confidence intervals and *P* values. For logarithmically transformed variables, *β* corresponds to percentage change. *P* values ≤0.05 were considered significant. Spearman’s correlations (*r*) were used to analyze associations between methylation levels. Using the SOLAR software (solar-eclipse-genetics.org), the influence of familiality (i.e., genetic and shared environmental effects combined) on DNA methylation and gene expression of *HIF3A* was estimated from a polygenic model as the proportion of the additive genetic variation and shared environmental effects on the total variation (the variance component approach). In the SOLAR models, familiality of *HIF3A* DNA methylation and gene expression was adjusted for age, sex, BMI, and HbA1c levels.

## Results

### Clinical characteristics

The study population had a wide age span (32–83 years) and varying levels of BMI (17.9–46.8 kg/m^2^) and glucose tolerance ranging from normal to overt T2D (Table 1). No significant differences between men and women were found for age, BMI, or HbA1c levels. Men had higher fasting circulating levels of glucose and insulin, but lower whole-body insulin sensitivity than women (Table 1).

**Table 1:** Anthropometrical and metabolic characteristics of the family population

	Men (*n =* 51)	Women (*n =* 86)	*P* value
Age (years)	53.8 ± 12.0	53.9 ± 10.7	*1.0*
Weight (kg)	90.7 ± 16.8	76.3 ± 15.9	*<0.0001*
Height (cm)	177.2 ± 7.9	165.8 ± 6.4	*<0.0001*
BMI (kg/m^2^)	28.8 ± 3.8	27.90 ± 6.0	*0.4*
Glucose tolerance status			
NGT (normal glucose tolerance)	26 (51 %)	61 (71 %)	
IFG (impaired fasting glucose)	3 (6 %)	4 (5 %)	
IGT (impaired glucose tolerance)	10 (20 %)	7 (8 %)	
T2D (type 2 diabetes mellitus)	12 (24 %)	14 (16 %)	
Fasting glucose (mmol/l)	6.9 ± 3.0	6.0 ± 2.1	*0.02*
Fasting insulin (pmol/l)	54.3 ± 35.5	46.2 ± 41.0	*0.05*
HbA1c (%)	5.8 ± 1.3	5.7 ± 1.0	*0.6*
Whole-body insulin sensitivity	6.27 ± 4.06	8.73 ± 5.29	*0.007*

All values are means ± SD. Fasting glucose, fasting insulin, and HbA1c values were logarithmically transformed to obtain normal distribution prior to analysis. *P* values were calculated in R 3.1.0 using a mixed linear model with family number as a random factor and sex, age, and BMI as a fixed factor. Glucose tolerance status: numbers (and percentage) of NGT, IFG, IGT, and T2D individuals

### *HIF3A* DNA methylation in the blood and SAT

**Fig. 2:**
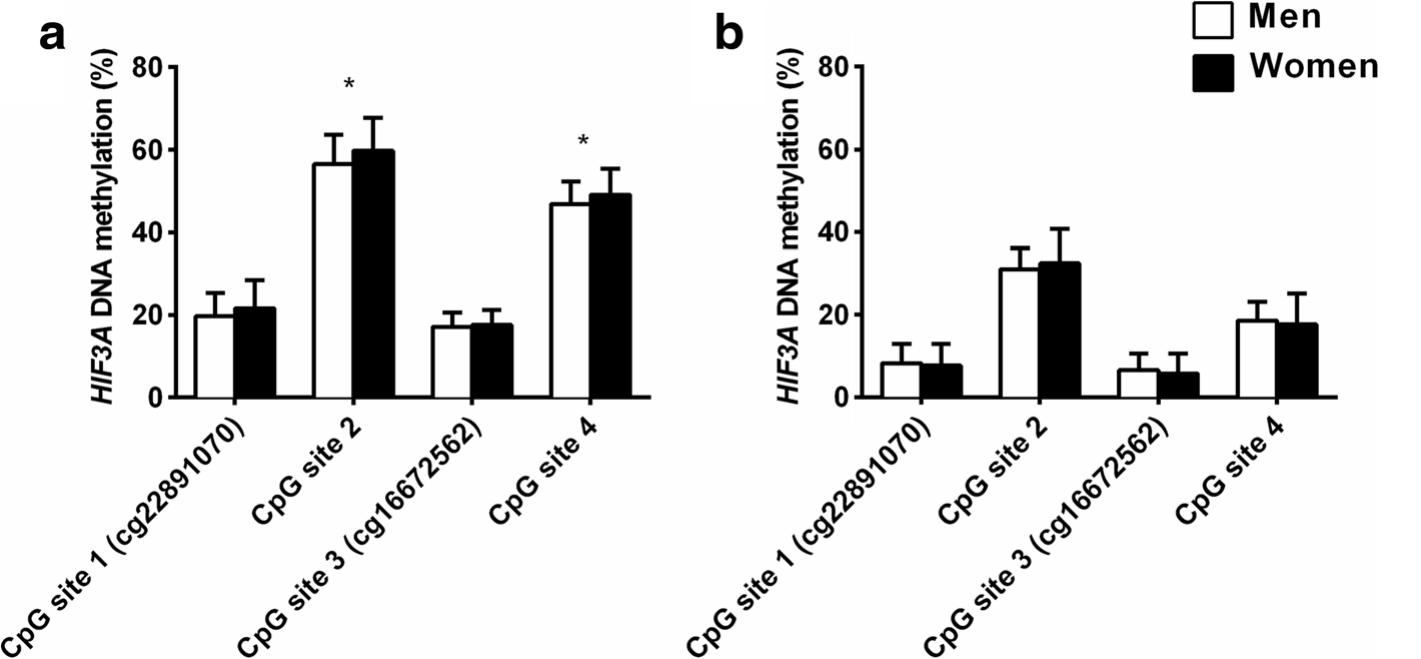
* ﻿HIF3A* promoter methylation levels of CpG sites 1 to 4 in blood (**a**) and SAT (**b**) in men and women. All values are mean ± SD. *P*-values for sex difference were calculated in R with family number as a random factor. *P<0.05, adjusted for BMI and age

DNA methylation levels on CpG sites 1 and 3 were lower compared to CpG sites 2 and 4 in the blood and SAT. A higher level of methylation was found on all sites in the blood compared to SAT (Fig. 2). Methylation levels on the different CpG sites associated with each other in both blood and SAT (blood, *r* = 0.39–0.69; SAT, *r* = SAT: 0.46–0.71; all *P* < 0.001), suggesting co-methylation of the sites investigated within the *HIF3A* region (Additional file 2: Figure S1 and Additional file 3: Figure S2). Furthermore, the DNA methylation level of CpG site 3 in the blood associated with the methylation level on the same site in SAT (*β* = 0.35 (0.06; 0.64) *P* = 0.02). The methylation level on the other three sites in the SAT and blood did not show any statistically significant associations (Additional file 4: Table S2).

Several individuals showed a methylation level of zero on CpG site 1 (16 individuals, 19 %) and CpG site 3 in SAT (26 individuals, 31 %).

There were no significant differences in *HIF3A* DNA methylation levels between T2D patients and non-diabetics.

### *HIF3A* methylation levels in the blood

*HIF3A* DNA methylation levels at CpG sites 2 and 4 in the blood were significantly higher in women compared to men (Fig. 2a). At CpG site 1, we found a significant positive association between BMI and DNA methylation (cg22891070: *β* = 0.263 (0.023; 0.502) *P* = 0.03). The association between BMI and *HIF3A* methylation at CpG site 1 persisted when T2D patients were excluded (*β* = 0.354 (0.111; 0.597) *P* = 0.004).

No significant associations between BMI and *HIF3A* methylation were detected in women or men separately, and we found no associations between whole-body insulin sensitivity and the level of *HIF3A* DNA methylation in the blood for any of the four examined methylation sites.

### *HIF3A* methylation levels in SAT

In SAT, we found no significant effect of sex on *HIF3A* DNA methylation (Fig. 2b).

Also, we found no significant association between BMI and *HIF3A* methylation in SAT in all individuals or in women or men separately for any of the four CpG sites. However, after exclusion of T2D individuals, methylation at CpG site 3 showed a significant association with BMI (*β* = 0.304 (0.006; 0.602) *P =* 0.05).

*HIF3A* DNA methylation in SAT did not show significant associations with the estimate of whole-body insulin sensitivity.

#### Association between DNA methylation and gene expression in SAT

DNA methylation levels of the four investigated CpG sites in the *HIF3A* promoter did not show any significant association with the *HIF3A* gene expression level in SAT.

### *HIF3A* mRNA expression

#### *HIF3A* gene expression in SAT

Gene expression of *HIF3A* in SAT was negatively associated with BMI in all individuals (*β* = −2.858 % (−5.351; −0.30) *P* = 0.030) and in women alone (*β* = −3.052(−5.918; −0.20) *P* = 0.039). This association was not found in SAT from men alone.

Whole-body insulin sensitivity was significantly associated with SAT *HIF3A* gene expression when corrected for sex, age, BMI, and HbA1c (*β* = 1.813 (0.483; 3.144) *P =* 0.008). The positive association between whole-body insulin sensitivity and *HIF3A* gene expression persisted after exclusion of T2D patients (*β* = 1.985 (0.398; 3.573) *P* = 0.014). When stratified for sex, the association was significant in men (*β* = 2.415 (0.843; 3.987) *P* = 0.003), but not in women (*P* = 0.12).

SAT *HIF3A* expression levels were not significantly different in men (0.82 ± 0.48) and women (0.74 ± 0.42) or between T2D patients (0.70 ± 0.64) and non-diabetics (0.79 ± 0.39).

#### *HIF3A* skeletal muscle gene expression

In skeletal muscle, the gene expression of *HIF3A* was similar in women (1.07 ± 0.48) and men (0.93 ± 0.32). *HIF3A* gene expression in skeletal muscle tissue was not significantly associated with BMI. The gene expression levels in skeletal muscle did not show any significant association with whole-body insulin sensitivity.

Skeletal muscle *HIF3A* expression levels were not significantly different in T2D patients (0.95 ± 0.39) and non-diabetics (1.04 ± 0.44).

Finally, the gene expression level of *HIF3A* in skeletal muscle was significantly associated with the gene expression level of *HIF3A* in SAT (*β* = 0.16 (0.027; 0.30) *P* = 0.017).

#### Familiality of DNA methylation and gene expression of *HIF3A*

*HIF3A* DNA methylation levels in the blood were significantly influenced by familiality on all sites with familiality effects between 51 and 64 % (Table 2). In SAT, the effect of familiality on *HIF3A* DNA methylation on CpG sites 1 and 2 showed borderline significance (Table 2). Gene expression of *HIF3A* in skeletal muscle, but not in SAT, was significantly influenced by familiality (*h*^2^ = 43 %, *P* = 0.016) (Table 2).

**Table 2:** Familiality (%) of *HIF3A* DNA methylation and gene expression

	Blood (*h* ^2^ %)	*P*	SAT (*h* ^2^ %)	*P*	Skeletal muscle (*h* ^2^ %)	*P*
*HIF3A* DNA methylation						
CpG 1 (cg22891070)	56 ± 25	*0.008*	55 ± 37	*0.08*	n.a.	
CpG 2	61 ± 27	*0.01*	44 ± 34	*0.09*	n.a.	
CpG 3 (cg16672562)	64 ± 24	*0.003*	34 ± 39	*0.19*	n.a.	
CpG 4	51 ± 26	*0.02*	41 ± 33	*0.10*	n.a.	
*HIF3A* gene expression	n.a.		12 ± 20	*0.26*	43 ± 21	*0.02*

Familiality estimates for the methylation and gene expression of *HIF3A* estimated using SOLAR software adjusted for age, sex, BMI, and HbA1c. Data are means ± SD

*n.a.* not available

## Discussion

In line with previous studies, our current Danish study of 137 first-degree relatives of T2D patients showed the same positive association between BMI and *HIF3A* DNA promoter methylation in the blood. This association was also present in SAT among non-diabetic participants. We also found an association between DNA methylation on one site in the blood and SAT from the same individuals, which is in line with the previous proposals of whole blood DNA methylation profiling as a marker for epigenetic changes in other human tissues [9, 16]. The methylation level was not associated with *HIF3A* gene expression in SAT. The gene expression level was negatively associated with BMI and interestingly also positively with insulin sensitivity independent of BMI. This finding suggests that SAT is a putative functional tissue for Hif3a with the derived potential implications for metabolic diseases.

Positive associations between BMI and *HIF3A* methylation in the blood have been demonstrated in men and women [16–18], whereas the association between BMI and *HIF3A* methylation in SAT only has been shown in females [9, 16]. We used a mixed-sex population, and we did not find any sex-specific difference in the association between BMI and DNA methylation in the blood or SAT when men and women were studied separately. The *HIF3A* DNA methylation level in the blood, however, was higher in women than in men at two of the four CpG sites.

By including individuals from the same families, we were able to estimate the influence of familiality (genetic and shared environment effects) on DNA methylation of *HIF3A*. Interestingly, DNA methylation levels in the blood showed a relatively high level of familiality (51–64 %), and the DNA methylation pattern in SAT showed borderline significant estimates of familiality. Hence, the heritability and thereby potential genetic influence on *HIF3A* DNA methylation in the blood is at the same magnitude as that seen for obesity and T2D [6, 22]. A recent epidemiological study suggested a strong influence of vitamins B_2_ and B_12_ intake on the methylation level of the *HIF3A* locus, providing an example of one, potentially among several, environmental factors influencing *HIF3A* DNA methylation levels [23].

The possible physiological importance of HIF3A in SAT was highlighted by our novel findings of significant negative associations between *HIF3A* expression in SAT and both BMI and whole-body insulin resistance. The association with BMI was predominantly driven by the female participants whereas the association with whole-body insulin sensitivity was significant in men, but not in women. Taken together, our results point to a regulation of SAT *HIF3A* expression by BMI that is most predominant in women. On the other hand, the putative impact of SAT *HIF3A* expression on glucose metabolism seems more pronounced in men. Even though T2D and non-diabetic individuals did not show alternating gene expression levels, the exclusion of T2D patients did not change the association between *HIF3A* mRNA expression levels in SAT and whole-body insulin sensitivity. This suggests that the association is present already in early stages of insulin resistance in peripheral tissues.

In line with our findings, it has previously been demonstrated that a lower expression level of *HIF3A* in adipocytes renders these cells less mature and less able to take up fatty acids [4] which could infer insulin resistance in peripheral tissues like skeletal muscle. No significant associations between muscle *HIF3A* expression and BMI or insulin sensitivity were demonstrated pointing towards SAT as a putative functional tissue for HIF3A.

We were not able to replicate the previously reported inverse association between a *HIF3A* promoter methylation and *HIF3A* gene expression in SAT [16]. Also, we found no significant effect of familiality on *HIF3A* expression in SAT. In the muscle, we demonstrated a significant effect of familiality on the *HIF3A* expression which suggests tissue differences of genetic regulation of *HIF3A* in the muscle and SAT. However, we found a positive association between *HIF3A* gene expression levels in the SAT and skeletal muscle suggesting some degree of co-regulation.

The number of participants in our study was lower than in the study by Dick et al. where the blood from 479 individuals of both genders and SAT from 635 women were included and this may explain why we could only just replicate the associations between BMI and *HIF3A* methylation. Methylation data from SAT were only available from a subset, and tissue from several individuals showed no methylation. This may have lowered the statistical power of our association analyses and familiality estimates in the SAT compared to the blood. A major strength of our study population is, however, the detailed phenotypic characterization that allowed an integrative physiological assessment, including insulin action, of the molecular findings. Causality, however, could not be examined in the present cross-sectional study design. Also, our study population had a higher risk of T2D by being first-degree relatives of T2D patients, and this may therefore not be representative of a random population. However, this fact may increase the relevance of the present findings for the understanding of the mechanisms underlying the development of T2D.

## Conclusions

Our findings are in line with the previously reported positive association between BMI and *HIF3A* methylation in the human blood. In the adipose tissue, however, we did not find significant positive associations between *HIF3A* methylation and BMI or whole-body insulin sensitivity. Furthermore, we provide novel insights into the tissue-specific regulation of *HIF3A* expression. Our findings point to a role of SAT, but not muscle, *HIF3A* expression for whole-body insulin sensitivity, with insulin-resistant individuals having the lowest expression levels. Further studies are needed to increase our understanding of the role of *HIF3A* in metabolic disease.

## Additional files

Additional file 1: Table S1. Total number of samples (*n*) with *HIF3A* DNA methylation and gene expression data after quality control. (PDF 374 kb) Additional file 2: Figure S1. Correlation between *HIF3A* DNA methylation levels at CpG sites 1 to 4 in blood. (PDF 67 kb) Additional file 3: Figure S2. Correlation between *HIF3A* DNA methylation levels at CpG sites 1 to 4 in subcutaneous adipose tissue. (PDF 126 kb) Additional file 4: Table S2. Associations between *HIF3A* DNA methylation levels in blood and subcutaneous adipose tissue. (PDF 175 kb)
