# Supplementary material for: DNA methylation and gene expression of HIF3A: cross-tissue validation and associations with BMI and insulin resistance
Source: Clin Epigenetics. 2016 Sep 2;8(1):89. doi: 10.1186/s13148-016-0258-6 (PMC5010678; doi:10.1186/s13148-016-0258-6)
Supplement: Additional file 1: Table S1. — Total number of samples (n) with HIF3A DNA methylation and gene expression data after quality control. (PDF 374 kb) [file 13148_2016_258_MOESM1_ESM.pdf]

Table S1: Total number of samples (n) with *HIF3A* DNA methylation and gene expression data after quality control

***HIF3A* DNA methylation data**	**CpG 1 (cg22891070)**	**CpG 2**	**CpG 3 (cg16672562)**	**CpG 4**
Blood (n)	108	108	105	107
SAT (n)	84	84	83	83
***HIF3A* mRNA expression data**				
SAT (n)	117			
Skeletal muscle (n)	120			

SAT: subcutaneous adipose tissue
