# Supplementary figures and images for: DNA methylation and gene expression of HIF3A: cross-tissue validation and associations with BMI and insulin resistance
Source: Clin Epigenetics. 2016 Sep 2;8(1):89. doi: 10.1186/s13148-016-0258-6 (PMC5010678; doi:10.1186/s13148-016-0258-6)

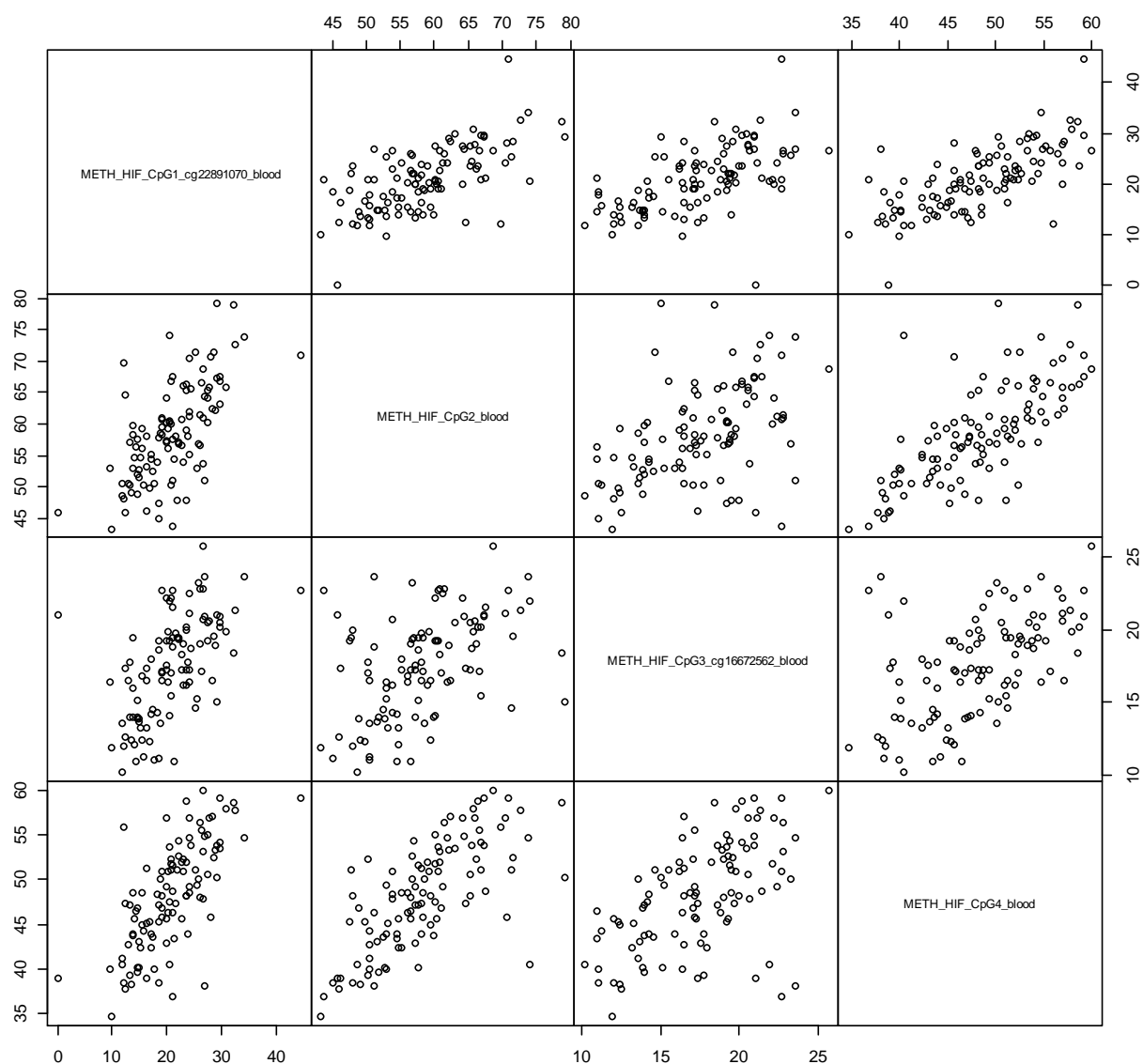

Supplement: Additional file 2: Figure S1. — Correlation between HIF3A DNA methylation levels at CpG sites 1 to 4 in blood. (PDF 67 kb) [file 13148_2016_258_MOESM2_ESM.pdf]

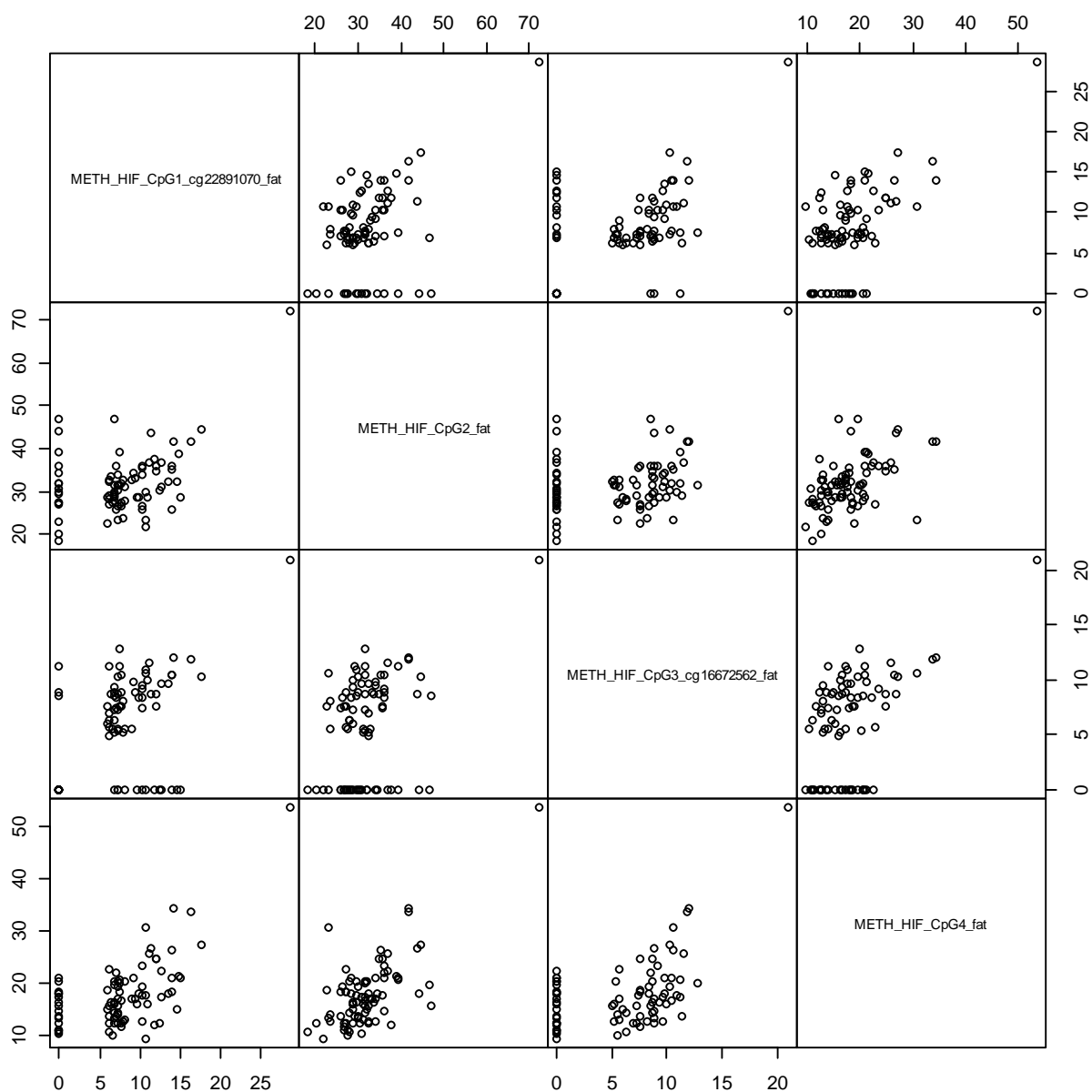

Supplement: Additional file 3: Figure S2. — Correlation between HIF3A DNA methylation levels at CpG sites 1 to 4 in subcutaneous adipose tissue. (PDF 126 kb) [file 13148_2016_258_MOESM3_ESM.pdf]
