# Supplementary material for: DNA methylation and gene expression of HIF3A: cross-tissue validation and associations with BMI and insulin resistance
Source: Clin Epigenetics. 2016 Sep 2;8(1):89. doi: 10.1186/s13148-016-0258-6 (PMC5010678; doi:10.1186/s13148-016-0258-6)
Supplement: Additional file 4: Table S2. — Associations between HIF3A DNA methylation levels in blood and subcutaneous adipose tissue. (PDF 175 kb) [file 13148_2016_258_MOESM4_ESM.pdf]

Table S2: Associations between *HIF3A* DNA methylation in blood and subcutaneous adipose tissue

***HIF3A* methylation**	**Blood vs. SAT**	***P***
CpG 1 (cg22891070)	0.111 (-0.042;0.2634)	0.15
CpG 2	0.075 (-0.110;0.261)	0.43
CpG 3 (cg16672562)	0.350 (0.057;0.642)	**0.02**
CpG 4	-0.023 (-0.220;0.173)	0.82

Values are effect estimates (β) and 95 % confidence intervals. Data were analysed with a linear mixed model with family number as a random factor. SAT: subcutaneous adipose tissue.
